# Proceedings from the CIH^LMU^ occupational safety and health symposium 2019 “Protecting workers’ health: global challenges and opportunities in work-related respiratory diseases”

**DOI:** 10.1186/s12919-020-00197-x

**Published:** 2020-12-02

**Authors:** Netsanet Workneh Gidi, Anna Suraya, Beatrice Mutayoba, Bernarda Espinoza, Bindiya Meggi, Issa Sabi, Jessica Michelle Guggenbuehl Noller, Kristina Schmieding, Nur Tukhanova, Martina Manhart, Arlett Heiber

**Affiliations:** 1 CIH-LMU Center for International Health, University Hospital, LMU Munich, Munich, Germany; 2 College of Public Health and Medical Sciences, Jimma University, Jimma, Ethiopia; 3 Occupational Safety and Health Department, Binawan University, Jakarta, Indonesia; 4 National Tuberculosis (TB) and Leprosy Control Program, Ministry of Health, Community Development, Gender, Elderly, and Children, Dar es Salaam, Tanzania; 5 Institute and Clinic for Occupational, Social and Environmental Medicine, University Hospital, LMU Munich, Munich, Germany; 6 Instituto Nacional de Saúde, Maputo, Mozambique; 7 Mbeya Medical Research Center, National Institute for Medical Research (NIMR), Mbeya, Tanzania; 8 Division of Infectious Diseases and Tropical Medicine, University Hospital, LMU Munich, Munich, Germany; 9 Asfendiyarov Kazakh National Medical University, Almaty, Kazakhstan

**Keywords:** Occupational safety, Occupational diseases, Work-related respiratory diseases, Protecting workers

## Abstract

The international CIH^LMU^ Occupational Safety and Health Symposium 2019 was held on 16th March, 2019 at the Ludwig-Maximilians-Universität Munich, Germany. About 60 participants from around the world representing occupational health and safety professionals, students, instructors from several institutions in Germany and abroad, attended the symposium.

The main objective of the symposium was to create awareness on global challenges and opportunities in work-related respiratory diseases. One keynote lecture and six presentations were made. While the keynote lecture addressed issues on occupational diseases in the twenty-first century, the six presentations were centered on: Prevention and control of work-related respiratory diseases, considerations; Occupational health and safety in Mining: Respiratory diseases; The prevention of TB among health workers is our collective responsibility; Compensation and prevention of occupational diseases and discussion on how artificial intelligence can support them: Overview of international approaches; Work-related Asthma: Evidence from high-income countries; and The role of imaging in the diagnosis of work- related respiratory diseases. A panel discussion was conducted following the presentations on the importance and challenges of data acquisition which is needed to have a realistic picture of the occupational safety and health status of workers at different levels. The current summary is an attempt to share the proceedings of the symposium.

## Background

Work-related respiratory diseases contribute for 70% of all occupational disease mortality [1]; these include interstitial or fibrotic lung diseases, hypersensitivity pneumonitis, lung cancer, lung infections, work-related Asthma, Chronic obstructive pulmonary disease (COPD), and bronchiolitis obliterans/airway destruction. These conditions are caused by inhalation of mineral dusts such as silica, asbestos, coal dust, and/or various metals and chemical gases and vapors [2].

Diagnosis of occupational diseases can be challenging as identification of an occupational cause for a disease requires exclusion of many other factors, which could potentially confound the presentation of a disease, resulting in discrepancy in the accuracy of diagnosis and underreporting [3, 4]. Similar to other medical conditions diagnosis of occupational respiratory illnesses is made based on history, physical examination, chest x-ray film, and pulmonary-function tests [5, 6].

Occupational exposure to asthmagens is associated with uncontrolled adult-onset asthma, though occupational risk factors are not clearly identified, which is essential to guide prevention approaches [7]. In addition to the suffering the victims experience, work related asthma symptoms are associated with significant professional, financial, and social consequences [8]. Work-related asthma and COPD are known to be linked with exposure to cleaning products. Information regarding risks and preventive measures need to be readily available for general population, professional and domestic cleaners [9, 10].

Carcinogens like asbestos have been identified to cause lung cancer with lifetime occupational exposure [11]. Ex-miners could develop exposure related diseases after long latency, and the sequelae of silica exposure regardless of the severity of the level of exposure remained one of the occupational health priorities, especially in a setting where concomitant HIV and TB infections are widespread [11].

The burden of occupational respiratory diseases is increasing worldwide, which necessitates the need for vigilant surveillance for development of public health policies, timely identification and interventions, with especial attention to high risk work places [12]. Occupational respiratory diseases are better addressed through primary preventive interventions, by measures taken to reduce exposure levels in the workplaces [13, 14].

### The CIH^LMU^ OSH symposium 2019

The CIH^LMU^ Occupational Safety and Health (OSH) Symposium 2019 addressed current global challenges in the diagnosis and management of work-related respiratory diseases, as well as the challenges health systems face in implementing interventions to prevent work-related respiratory diseases. The symposium was attended by about 60 participants from academia, research institutions, the private sector, as well as policy makers and health care providers.

The objectives of the symposium were to increase awareness and knowledge of the current condition of occupational health worldwide, to discuss the challenges and opportunities that health systems face, and to facilitate dialogue on effective interventions and strategies to prevent occupational respiratory diseases.

The symposium was officiated by Prof. Dr. Michael Hoelscher, Director of the Division of Infectious Diseases and Tropical Medicine, University Hospital, LMU Munich, Germany, who welcomed all participants and speakers to open the symposium. Dr. Elizabet Paunovic, former head of the World Health Organization (WHO) European Centre for Environment and Health (Bonn, Germany), gave a key note speech on occupational diseases in the twenty-first century, followed by the detailed presentations on prevention and control of work-related respiratory diseases, with due focus on high risk settings. A summary of each presentation based on the records and issues discussed are presented below.

### Presentation summaries

#### Keynote lecture: occupational diseases in the twenty-first century

Dr. Elizabet Paunovic, former Head of the World Health Organization (WHO) European Centre for Environment and Health (Bonn, Germany).

Globally, more than 2.3 million people die each year from occupational accidents or work-related diseases. The difference between occupational disease and a work-related disease has been a point of discussion among occupational safety and health specialists. An occupational disease is a case of disease recognised by the national authorities as being caused by a factor at work, linked with the insurance during legally regulated type of work. A work-related disease comprises occupational diseases and other diseases, whose etiology is partly affected by work-related factors (like musculoskeletal diseases, cardiovascular diseases, many respiratory diseases and mental disorders).

Occupational diseases are largely preventable; therefore, health workers in line with the principle of sound occupational health services should give emphasis for prevention, protection, and promotion of workers’ health. Similarly, protection of access to work, entitlement to compensation, health insurance benefits, and social protection need to be addressed according to the international laws in place. There is significant underreporting occupational diseases, for example, on the global level, only 82 out of 120 countries have a national registry on occupational diseases and in Europe 40 out of 48.

As it was presented at the Hague Global Conference 2011, the public health approach to workers’ health prompted by WHO goes well beyond the concept of ‘occupational health’, and considers workers’ health as part of general health and life. Health systems should facilitate local strategies to meet workers’ health needs, in moving towards universal coverage; those at greatest risk or having greatest needs should be targeted first. When developing policies about workers’ health all relevant stakeholders should be involved; training in health and work should be part of all health care professional training, and empowerment of workers and the encouragement of decision-makers are critical for the promotion of the health and safety of workers. Incorporation of workers’ health into other policies is another important approach to alleviate the burden of the disease. Some of these policies include, relevant environmental policies and initiatives; strategic approach to international chemicals management (SAICM); multilateral environmental agreements: Rotterdam, Basel, Stockholm, and Minamata conventions; emergency preparedness and response; climate change mitigation and adaptation strategies, sectoral policies for branches with highest health risks.

Regarding potential burden of the diseases, estimations are based on research data because there are no exposure data available. In Europe, ergonomic and noise hazards are higher and carcinogens lower compared with the world population. Many serious occupational diseases have a long period of ‘latency’, some up to 30 years, between exposure and development of ill health and/or disease, making the links even more difficult to establish, for example, lung carcinoma caused by asbestos exposure. During the last years, developed countries ban the use of asbestos. However, nowadays they are reaching the peak of lung cancer due to exposure to asbestos. In developing countries, they do not admit the association between lung cancer and asbestos exposure because they do not have yet a high incidence of lung cancer patients. This also means that after recognizing the problem and making changes in working practices to reduce exposure, there may be a long delay before a reduction in the causes of ill health and death are seen.

#### Prevention and control of work-related respiratory diseases, considerations

Prof. Dr. Frank van Dijk, emeritus professor in Occupational Health at the Academic Medical Center in Amsterdam.

Only 15% of workers in developing countries and 50–90% of workers in most industrialized countries have access to OSH professionals or OH services [15]. Primary and community health care cover majority of the global population, including almost two billion workers [16]. Therefore integration of occupational health in the primary care setting benefits of all workers and their families.

Member states shall establish, maintain, progressively develop and periodically review a national system for occupational safety and health, in consultation with the most representative organizations of employers and workers. The International Labour Organization (ILO) Convention number 187 recommended that the national system for occupational safety and health shall include among others: national tripartite advisory body; information and advisory services on OSH; OSH training; OSH services in accordance with national law and practice; research on OSH; a mechanism for the collection and analysis of data on occupational injuries and diseases, taking into account relevant ILO instruments; provisions for collaboration with relevant insurance or social security schemes covering occupational injuries and diseases; support mechanisms for a progressive improvement of OSH in micro-enterprises, in small and medium-sized enterprises and in the informal economy.

Occupational safety and health services are structured in different health systems around the world. For example, Indonesia has 5 occupational health centers, 30% of the community health centers are trained in OSH and 5.000 Occupational health post use volunteers [17]. Iran has integrated OSH in primary health care [18]. Thailand started to deliver basic OSH services in primary care units in 2004. Currently, a significant number of primary care units can provide OSH services in the country [19]. Turkey developed a project in the Community Health Centres in 2016 to provide OSH care for small companies, self-employed and the informal sector [20].

There are limited studies about the use of the internet as a tool of information for workers. There is a demand for information, education and occupational health care, that are tailor-made, evidence-based, practice-based, feasible, widely accessible and affordable [21, 22]. The coverage should be wide including informal workers, self-employed and small enterprises. Reliable apps, websites, online videos, Q&A’s, e- and blended learning have a great potential [23, 24]. National governments are responsible for the structural development of primary health care activities for workers health, inclusive financial resources, education and support by preventive OSH-experts and clinical referral centers.

#### Occupational health and safety in mining: respiratory diseases

Dr. Erik Jørs, Chair MinOSH, Clinic of Occupational and Environmental Medicine, University of Southern Denmark, Odense, Denmark.

About 9 million people are involved in formal mining companies, and about 20–30 million people in informal mining, among them many women and children. The four most profitable minerals are coal, copper, gold and iron. Small-scale sectors produce 20% of global gold supply, 25% of tin, 26% of tantalum, and 20% of diamonds. Occupational diseases in Mining include accidents, silicosis, coal workers’ pneumoconiosis, asbestosis, cancers, psychosocial stress, hearing loss, ergonomic diseases, poisonings etc.

Silicosis is the most common occupational disease around the world. Types of occupations with silica dust exposure are mining, construction, tunnelling, sand blasting, stone crushing etc. In the World Health Report of 2002, WHO estimated that pneumoconiosis costs 30,000 lives per year [25]. Diseases associated with respirable crystalline silica (quarts, SiO_2_) seen in miners include chronic silicosis (develops after 10–20 years), accelerated silicosis (takes 3–10 years to develop), chronic bronchitis, emphysema, pulmonary and extra-pulmonary TB, lung cancer, scleroderma, systemic lupus erythromatosis, rheumatoid arthritis, and renal diseases. Host factors influence individual susceptibility, and smaller particles increase the fibrogenicity of the dust and disease can develop years after exposure has stopped.

Permissible exposure limit for crystalline silica is in a range from 0.05 to 0.2 mg/m^3^ in the EU and the US, the risk of silicosis following a lifetime of exposure at 0.05 mg/m^3^ is likely to be 20–40%. Prevalence of silicosis as reported in literatures: among gold miners in Ecuador 11% of them were found to have silicosis, gold miners in South Africa during 2004–2009 showed mean prevalences of 5,7% with a sharp increase depending on the number of years in mining, in Brazil wells dug by hand through rock with very high quartz content (97%) resulted in a silicosis prevalence of 26%.

Silicosis is a risk factor for TB, miners in formal sector experience incidence rates of TB 3–4 times greater than the background population. HIV and silicosis coexistance increases the risk of developing TB by about 15 times compared to the non-exposed population. The ILO/WHO program for the elimination of Silicosis was established in 1997 with the objective to have silicosis eliminated by 2030.

Coal workers lung diseases are anthracosis (asymptomatic accumulation of carbon without silicosis), CWP (coal workers pneumoconiosis) with only small radiographic opacities or larger opacities > 10 mm called progressive massive fibrosis, COPD and lung cancer. The prevalence of CWP in USA and china is 3.2 and 6% [26]. Asbestos is mined and used due to its physical properties, such as sound absorption, tensile strength, resistance to fire, heat, chemical and electrical damage. Asbestos-related lung cancer, mesothelioma, and asbestosis (asbestos pneumoconiosis) resulted in 107.000 deaths from occupational exposure in 2004 according to WHO estimates. WHO, ILO and International Commission on Occupational Health (ICOH) have declared a total ban on asbestos by 2014. Fifty countries have banned mining and/or uses of asbestos, exceptions are USA, China, Russia, India, Brazil and Indonesia. In accordance with ICOH Call for Action, silica dust controls should be explicitly included in national TB prevention strategies; dust control should be included in all activities to improve occupational health in mining, e.g. in mercury reduction programs in small-scale gold mining; international lenders (e.g. World Bank) should incorporate strict dust controls into infrastructure (construction) and mining projects; governments and global health community has to follow up implementation of the above recommendations. The United Nations (UN) political declaration on TB, ‘United to end TB: an urgent global response to a global epidemic’ also focuses on ‘Preventing TB amongst Silica-Exposed Workers’ and the need to control dust and silicosis as an essential part of TB control programme at company, national and regional level.

Dust prevention strategies include control at the source, control in the transmission path, measures related to the worker, and measures related to the work environment such as good housekeeping, storage, labeling warning signs and restricted areas, environmental monitoring/alarm system. Preventing silicosis by investing in engineering controls to reduce silica dust emissions is one of the most cost effective public health interventions in the workplace; dust controls are more cost effective than investing in public health programs to identify and treat cases of TB, dust reduction also prevents silicosis, lung cancer, and other silica-related diseases.

#### The prevention of tuberculosis among health workers is our collective responsibility

Dr. Sophia Kisting, Occupational Medicine Specialist, Cape Town, South Africa.

TB is a major occupational risk for health workers especially in high burden countries. World Leaders endorsed the UN Political Declaration on TB on September 26, 2018; “United to End TB: An Urgent Global Response to a Global Epidemic” [27]. The Declaration states “We, Heads of State and Governments and representatives of States and Governments, assembled at the United Nations in New York, with a dedicated focus for the first time on the global TB epidemic, reaffirm our commitment to end the TB epidemic globally by 2030 in line with the Sustainable Development Goals target and commit to end the epidemic in all countries, and pledge to provide leadership and to work together to accelerate national and global collective actions”. What impact does it have in life of nurses working in various parts of the world? Declaration is important, putting it in to practice needs commitment of authorities.

Regarding challenges health workers face in high TB burden countries, a higher risk of developing TB and hospitalization for multi-drug resistant TB has been reported [28]. Gender inclusive approach towards all workplace preventive interventions is mentioned important as women comprise over 70% of the health workforce in many countries. A high rate of TB among health workers has a negative impact on human resources for health, given the estimated needs-based shortage of health workers globally, about 17.4 million.

The lung destruction caused by TB can lead to severe lung disability and heart failure. TB treatment as well has serious adverse effects, such as hearing loss, vision loss, liver damage and nerve dysfunction. And people who have had TB are about 3 times more likely to develop TB than other people who never had the disease in the past.

Challenges to preventive strategies in low resource, high TB burden settings include large numbers of undiagnosed TB patients, health staff shortages, delays in diagnosis and treatment of patients, lack of OSH expertise and staff uncertainty regarding confidentiality and job security, paralyzing effect of TB stigma and discrimination and inadequate data collection and relevant research. Effective prevention approaches include primary prevention, triad of administrative controls, engineering controls and adequate respiratory protection; augmented by secondary prevention measures such as screening and treatment for both latent and active TB infection in health workers.

However, infection control and OSH structures are fragmented and poorly resourced in resource poor settings; more research is urgently needed in low income, high TB burden settings. There is a need for developing and implementing OSH and Infection prevention and control (IPC) workplace preventive practices, and supporting policies to strengthen prevention, training and raising awareness. Early treatment is a major step forward for prevention. International collaboration is highly needed for research, research mentorship and greater skills development in OSH.

The speaker stated that the presentation is not a lament about the TB problem in Africa, rather attempts to give recognition to our interdependence, the interconnectedness of our world and our shared humanity to address major health problems and asked the audience to endorse the ICOH TB statements.

#### Compensation and prevention of occupational diseases, and discussion on how artificial intelligence can support them: overview of international approaches

Dr. Hector Upegui, worldwide market development executive and associate chief health officer at IBM Watson health.

Occupational diseases demand large amount of time for case analysis, evidence finding and diagnosis. Furthermore, diagnosis includes not just the medical process but also legal analysis for causation, as well as for properly identifying the respective entitlement of the person to receive benefits in cash and in kind. All these together with additional components for prevention are especially intensive in time, knowledge and money just to mention some. Computers with cognitive computing capabilities can read, understand, reason, learn and interact with humans and with other machines. These capabilities can be used to free time for practitioners, claims adjusters and patients among others. Modern technology can be used to integrate large amount of data including images, voice, video and audio from different type of sources.

Occupational Health systems, including social security dealing with occupational diseases, should also be taking advantage of the fact that people are now interested in getting their own data, for example by accessing their own labor history, risk exposure during their working life, claim status or health evolution. Data analysis to take real life actions can be offered in an easier and digestible way, adjusted to different roles and interests for the different stakeholders. Artificial intelligence, advanced analytics, can make this happen and it’s already taking place in some other sectors, like banking insurance, retail, just to mention some.

Whatever initiative is taken with artificial intelligence, it must respect three principles proposed already by International Business Machines Corporation (IBM): Purpose, transparency and skills. A clear purpose on what’s the ultimate goal of applying artificial intelligence to a specific problem, assuring that data stays with the owner of that data, avoiding “black-box” algorithms and being sure that machine is properly trained by people being experts on their field, is paramount in applying this new technology.

Occupational Health systems are fragmented and this fragmentation makes it difficult to fluently implement processes. For example, an occupational disease caused by a very long term exposure to a toxic agent involves social security insurance company (public or private), family physician, occupational health and safety specialist, employer, family members, lawyers, judges, hospitals where service is being provided, etc., all of them with different access to information and different understandings of risk, diseases and policies. This is a great challenge for all involved, especially for the worker claiming an occupational disease, as this one is ultimately the one “integrating” the system by repeating the story and providing documentation to the different stakeholders involved in the case.

IBM Watson, the artificial intelligence machine from IBM participated in Jeopardy and won. In doing that IBM Watson answered difficult questions by reading more than 200 million pages in about 3 s [29]. A good example on how this technology could be practically used in an occupational health setting is by helping the physician for example, to prepare the case. The computer reads all previous data of a patient and gives back information to help the doctor analyze important facts and values that are key to the occupational disease diagnosis. The technology can also answer questions on available evidence-based medicine, to pick related scientific findings supporting or not a hypothesis of the doctor regarding the relationship of a concrete occupational risk and the disease. This can help the physician and the patient save time and give additional elements to support the doctor with a more accurate diagnostic. Finally, we should all bear in mind, that artificial intelligence and in the case of IBM Watson, is trained by people, what shows us, that more and more in the future this technology would need new skills from all of us professionals in the healthcare area.

#### Work-related asthma: evidence from high-income countries

Dr. Tobias Weinmann, Epidemiologist and post-doctoral researcher, Institute of Occupational, Social and Environmental Medicine, University Hospital, LMU Munich, Germany.

Work-related asthma (WRA) is mostly reported from North America and Europe and often considered as a ‘western disease’ attributable to a ‘western’ lifestyle. However, the primary evidence from studies done in high-income countries should be valid and applicable to low-income countries, as underdiagnoses and underreporting is likely to be the reason for the low rate of WRA in low-income countries.

WRA can be further classified into two sub-groups: work-exacerbated asthma (WEA) and occupational asthma caused by work (OA). WEA accounts for 21.5% of adults with asthma, occurs when pre-existing or concurrent asthma is worsened by conditions in the workplace, such as from exposure to chemicals, dusts, second-hand smoke, allergens and emotional stress, among others. Patients with WEA usually have compromised quality of life and require more medical care than patients with asthma unrelated to work, so WEA causes a considerable public health impact. OA is defined as a new onset of asthma caused by workplace exposures and accounts for around 15% of asthma cases. The two sub-groups of OA: Sensitizer-induced OA and Irritant-induced OA. Sensitizer-induced OA more commonly understood as ‘allergic asthma’, occurs when a sensitizer, or agent, induces asthma through a specific immunologic response. On the other hand, irritant-induced OA has two states: acute, causing reactive airways dysfunction syndrome (RADS), and a chronic state, causing chronic bronchitis, asthma, and asthma-like symptoms. RADS can occur after a single high-level exposure – an example of this was the highly irritating dust after the collapse of the World Trade Center. Group of workers such as cleaners, nurses, textile workers, and potroom workers are at risk for chronic irritant-induced OA, due to the long-term exposure to chemicals at work. Several studies have proven the associations between occupational exposure to pesticides and respiratory symptoms in farming, and cleaning products and asthma.

Prevention of exposure should be considered in the management of WRA. The study by Allmers et al. (2002) has illustrated how education and simple interventions can make a large difference in the health of workers [30]. Allmers and colleagues documented the rapid decline of occupational asthma cases in German health workers after the introduction of low-protein, powder free natural rubber latex (NRL) gloves in the mid 1990s.

Unfortunately, many manufacturers of cleaning products have disregarded research conclusions and policy suggestions to make products safer. Consumer and public interest groups can educate workers, for example cleaners, to not mix products, and to minimize exposure to harsh cleaning products such as bleach, ammonia and disinfectants. Along these lines, workers and public health officials can help to influence policy and industry by showing the economic advantages of safer products. Workers will require less medical leave if they work with safer materials. In regard to farming, farmers could benefit from improved ventilation in animal houses, which could also lead to less use of antibiotics for animals.

#### The role of imaging in the diagnosis of work-related respiratory diseases

Prof. Dr. Kurt Georg Hering, consultant radiologist, visiting professor of Kochi University and a member of the ILO-Panel.

Some work-related respiratory diseases have characteristic radiologic features suggesting the diagnosis. However, definite diagnosis requires combination of occupational history, medical examination and imaging features, interpretation of radiologists is needed for accurate diagnosis. In OSH there is a need for radiologists who are specialized in occupational diseases diagnosis. The chest X-ray (CXR) only serves the role of an epidemiological instrument, since it only helps finding the disease. The CXR does not find the correlation between lung function and compensation.

Changes in the lung caused by the retention of and the reaction to inhaled dust (organic as well as inorganic) are classified into two, those with Granuloma (e.g. Silicosis) and without Granuloma (e.g. asbestosis). The lack of visible vessels distribution seen in a normal x-ray can be a potential sign of fibrosis. Distribution of pulmonary diseases can suggest the likely diagnosis, conditions with upper zones preference include, Silicosis, TB, Respiratory bronchiolitis interstitial lung disease (RB-ILD), Smoking related emphysema, Langerhans cell histiocytosis, mucoviscidosis. Sarcoidosis is known to show middle zones preference, while lower zones preference is observed with usual interstitial pneumonia (UIP)-Pattern idiopathic pulmonary fibrosis (IPF), asbestosis, collagenosis, nonspecific interstitial pneumonia (NSIP), bronchiolitis obliterans; no predominance is expected with hypersensitivity pneumonitis or extrinsic allergic alveolitis (HP/EAA) and lymphangiosis.

Chest radiography is an important tool for clinical evaluation of pulmonary diseases, especially infectious lung diseases, diffuse lung diseases or the interstitial lung diseases and neoplastic diseases. Chest radiographs are useful in clinical care, assisting in both diagnosis and evaluating response to therapy. However, pattern recognition on CXR requires important clinical skill for evaluation of acute and chronic lung diseases. Chest Computed Tomography (CT), especially as High-resolution CT (HRCT), provides significantly improved information and is essential for a definitive diagnosis. Nevertheless, the plain chest films cannot be replaced, also for reasons of availability and the resulting costs.

**Fig. 1:**
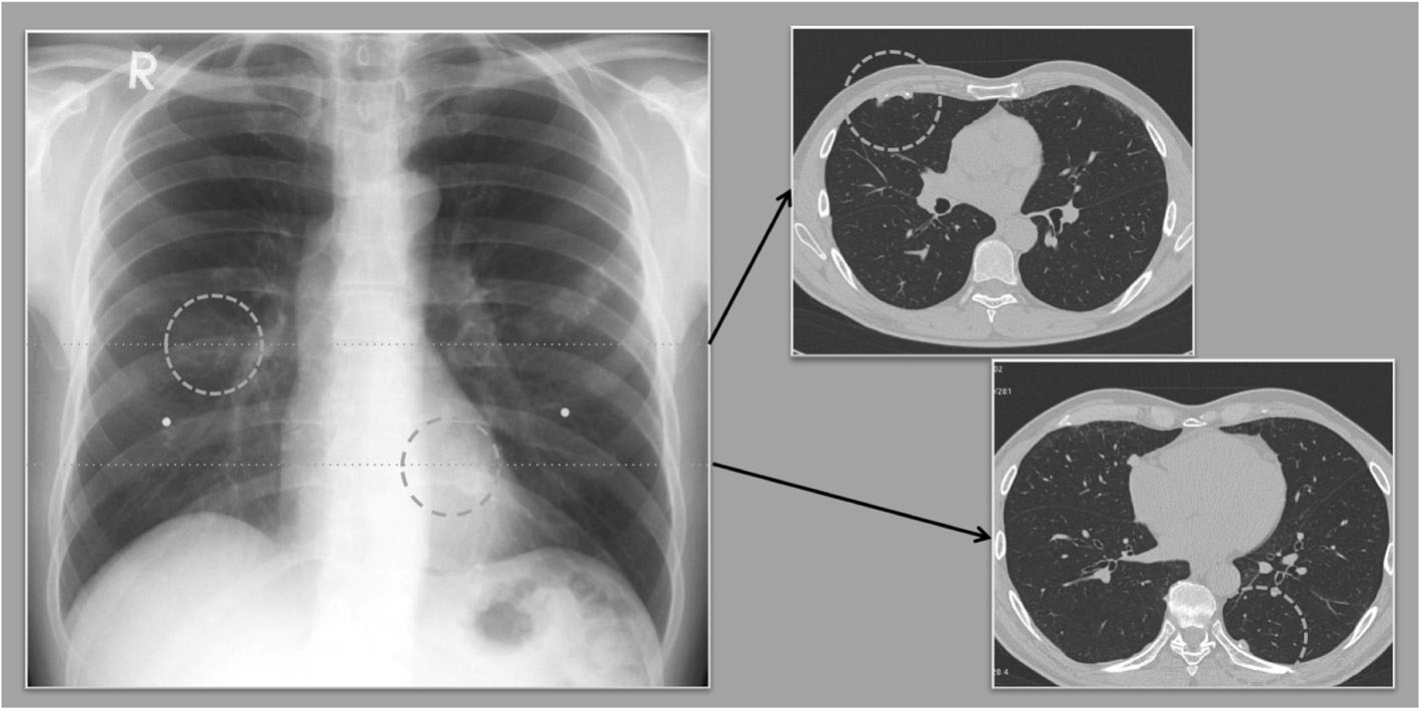
Comparison analog/digital Chest Radiograph versus Computed Tomography, findings on the pleura are overlooked or in face-on projection confused with tumor (Source: Prof. Dr. Kurt Georg Hering)

The finding of pleura can confuse with presence of a tumor, requiring CT scan to differentiate between the two (Fig. 1). In order to be able to compare the localization of a finding in the chest film with the computed tomographic image, the 3 zones of the ILO-classification (upper, middle and lower zones) have to be transferred virtually to the regions in the CT.

In conclusion, imaging in the diagnosis of work-related respiratory diseases plays a role as tool for epidemiology and monitoring. Chest X-ray as a basic study is available everywhere and affordable. Low Dose-Vol-CT is essential for the plausibility of diagnosis. Quality management program is essential for workplace assessment, personal medical and professional history, monitoring of the quality of radiological examination and diagnosis. Radiographic Imaging has limitations, especially without HRCT, as it is not diagnostic gold standard; airway disorders are not always seen, functional impairment not well evaluated or assessed and cannot provide certainty about the etiology of observed findings due to limited lung response patterns.

### Summary of the panel discussion

Moderated by Dr. Elizabet Paunovic, former head of WHO European Centre for Environment and Health (Bonn, Germany).

The panel consisting of all speakers of the day was asked to comment on challenges related to the collection of data relevant for occupational safety and health and the set of data that should be collected (reported) in order to get the realistic picture of the occupational safety and health status of workers at different levels (e.g. enterprise, factory, district, country, an area of work, etc.). The following important challenges and gaps were discussed.

- There are three main sources of OSH data: academic, surveillance and compensation, which are fragmented, making it difficult to have a reliable source of information without bias.
- Academia research data is crucial; however, many academic institutions do not utilize their data to influence policy makers. They keep the data for education purpose only. There is a fear of that data to be used to claim compensations. There is a need of publishing data in order to convince the policy makers about a burden of the occupational disease, cost related to prevention versus compensation.
- Monitoring of data on OSH is limited to those diseases which are known to be problematic and already identified as occupational disease. However, open platforms are needed to ensure detection of new OSH diseases.
- National Statistics are incomplete and not directed, leading to underreporting; many countries do not accept they have occupational disease, as there is no surveillance in place to capture the conditions as occupational diseases.
- Compensation statistics usually underreport the burden of occupational diseases that actual exists. Not all people who had or have occupational disease will go through the compensation process.
- To have a surveillance model that actually catches the true occupational disease burden is not easy but doable. For a surveillance system to work well there is a need of guidelines in all levels including at primary health care level, training of health professionals in how to identify potential occupational diseases, reference system in place to help the management of the cases, better communication between radiologists, pulmonologists and OSH specialist and education of the workers for them to know the risks they are exposed in their work.

## Conclusion

The burden of occupational respiratory diseases in the world is increasing. Less than one fifth of workers in the world have access to OSH professionals or occupational health services. Underdiagnosis and reporting is a challenge, especially from low-income countries. Occupational diseases surveillance system is fragmented and do not exist in some countries. Occupational diseases are largely preventable; responsible authorities and policy makers at country level and international organizations should give emphasis on surveillance of extent of the problem, monitoring the preventive measures taken by manufacturers and high-risk workplaces, and adequate care of the victims.

## Abbreviations

CIH^LMU^: Center for international health at Ludwig-Maximilians-universität München
COPD: Chronic obstructive pulmonary disease
CWP: Coal workers pneumoconiosis
HP/EAA: Hypersensitivity pneumonitis or extrinsic allergic alveolitis
HRCT: High-resolution CT
ICOH: International commission on occupational health
ILO: International labour organization
IPC: Infection prevention and control
IPF: Idiopathic pulmonary fibrosis
OA: Occupational asthma caused by work
OSH: Occupational safety and health
NRL: Natural rubber latex
RADS: Reactive airways dysfunction syndrome
RB-ILD: Respiratory bronchiolitis interstitial lung disease
SAICM: Strategic approach to international chemicals management
TB: Tuberculosis
UIP: Usual interstitial pneumonia
UN: United Nations
WEA: Work-exacerbated asthma
WHO: World health organization
WRA: Work-related asthma
